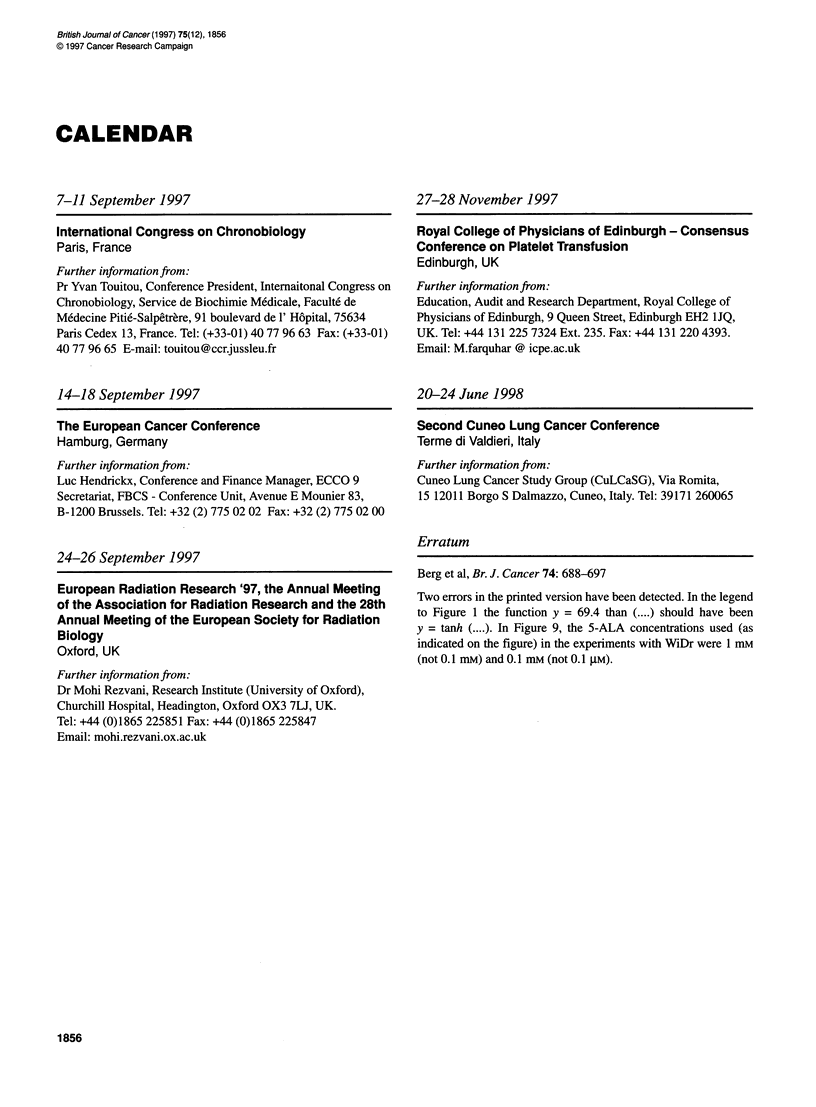# Calendar

**Published:** 1997

**Authors:** 


*British Journal of Cancer* (1997) **75**(12), 1856
© 1997 Cancer Research Campaign

### CALENDAR

### *7–11 September 1997*

**International Congress on Chronobiology**
Paris, France

*Further information from:*
Pr Yvan Touitou, Conference President, Internaitonal Congress on Chronobiology, Service de Biochimie Médicale, Faculté de Médecine Pitié-Salpêtrère, 91 boulevard de l' Hôpital, 75634 Paris Cedex 13, France. Tel: (+33-01) 40 77 96 63 Fax: (+33-01) 40 77 96 65 E-mail: touitou@ccr.jussleu.fr

### *14–18 September 1997*

**The European Cancer Conference**
Hamburg, Germany

*Further information from:*
Luc Hendrickx, Conference and Finance Manager, ECCO 9 Secretariat, FBCS - Conference Unit, Avenue E Mounier 83, B-1200 Brussels. Tel: +32 (2) 775 02 02 Fax: +32 (2) 775 02 00

### *24–26 September 1997*

**European Radiation Research '97, the Annual Meeting of the Association for Radiation Research and the 28th Annual Meeting of the European Society for Radiation Biology**
Oxford, UK

*Further information from:*
Dr Mohi Rezvani, Research Institute (University of Oxford), Churchill Hospital, Headington, Oxford OX3 7LJ, UK.
Tel: +44 (0)1865 225851 Fax: +44 (0)1865 225847
Email: mohi.rezvani.ox.ac.uk

### *27–28 November 1997*

**Royal College of Physicians of Edinburgh – Consensus Conference on Platelet Transfusion**
Edinburgh, UK

*Further information from:*
Education, Audit and Research Department, Royal College of Physicians of Edinburgh, 9 Queen Street, Edinburgh EH2 1JQ, UK. Tel: +44 131 225 7324 Ext. 235. Fax: +44 131 220 4393.
Email: M.farquhar @ icpe.ac.uk

### *20–24 June 1998*

**Second Cuneo Lung Cancer Conference**
Terme di Valdieri, Italy

*Further information from:*
Cuneo Lung Cancer Study Group (CuLCaSG), Via Romita, 15 12011 Borgo S Dalmazzo, Cuneo, Italy. Tel: 39171 260065